# Data on the UV filtering and radical scavenging capacity of the bitter masking flavanone Eriodictyol

**DOI:** 10.1016/j.dib.2018.08.149

**Published:** 2018-08-31

**Authors:** Vijisha K. Rajan, Shameera Ahamed T.K., K. Muraleedharan

**Affiliations:** Department of Chemistry, University of Calicut, Malappuram 673635, India

## Abstract

A computational analysis of UV filtering and radical scavenging capacity of a flavanone, Eriodictyol has been performed under DFT-B3LYP/6–31+ G (d, p). Eriodictyol is nontoxic and nonirritant bitter masker used in wine and can be used for photo protection due to its potential UV filtering and radical scavenging capacity. The compound has an absorbance in the UV-A and UV-B region of electromagnetic spectrum, it can be used as a potential UV filter in sunscreen lotions and other cosmetic products. Eriodictyol is a potent antioxidant than the most commonly studied Quercetin. The most active site in the compound is 3׳ position and is confirmed by NPA, NBO and pKa value analysis. The BDE values follow the order 3′<4′<7<5. The major transitions in the UV–visible spectrum of Eriodictyol are between HOMO and HOMO-1 with LUMO level and are well explained by NBO–NLMO tool in G09.

**Specifications Table**

**Table:** 

Subject area	*Chemistry*
More specific subject area	*Computational chemistry*
Type of data	*Table, image; word document*
How data was acquired	*Computational analysis through results obtained from structural optimizations by using tools in Gaussian 09 software package like TDDFT, NBO-NLMO,NPA, etc,.*
Data format	*Analyzed*
Experimental factors	*No experimental data, all results are computed.*
Experimental features	–
Data source location	*All data were computed through Gaussian 09 software package*
Data accessibility	*Data is with this article*
Related research article	*Vijisha. K. Rajan, Shameera Ahamed T.K, K. Muraleedharan: Studies on the UV filtering and radical scavenging capacity of the bitter masking flavanone Eriodictyol: JPHOTOBIOL: 2018. (in press)*

**Value of the data**

• Eriodictyol can be used as potential UV filter in sunscreen lotions and other cosmetic products.
• Eriodictyol is a potential antioxidant than the most commonly studied Quercetin.
• The pKa values of all the possible sites are computed on the basis of free energy values.
• Its BDE values follow the order 3′<4′<7<5 position in the flavanone ring.
• Major transitions in the UV–visible spectrum are between HOMO and HOMO-1 with LUMO.
• The UV filtering capacity can be well explained by the combined NBO–NLMO study.

## 1 Data

**Fig. 1:**
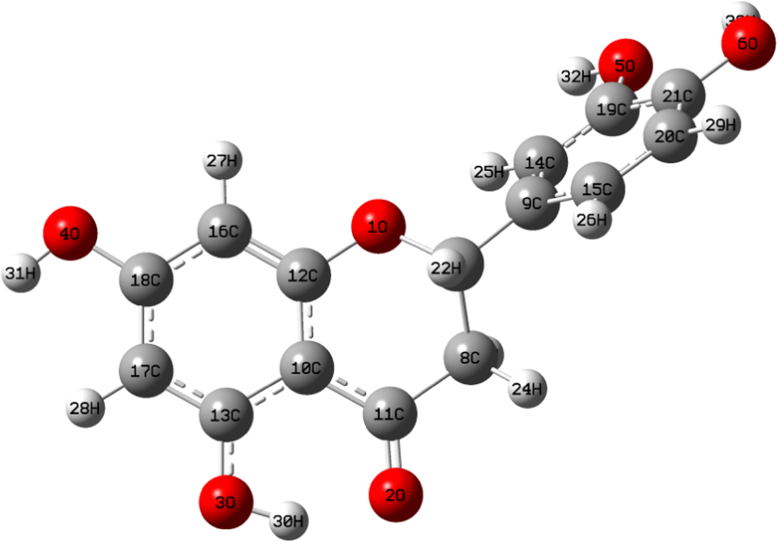
Numbering in the stable conformer of Eriodictyol.

The data include the structure and numbering of the optimized stable conformer of a flavanone called Eriodictyol (Fig. 1). A Density Functional Theory (DFT) based evaluation of the UV filtering and radical scavenging capacity of Eriodictyol has been described. All the computational calculations are carried out through Gaussian 09 software package and the Gaussview 5 graphical user interface [1], [2]. The optimized structure contains a hydrogen bond between the carbonyl oxygen and the H30 at position 5 in ring A. the molecule has –OH groups in ring A and B and the conjugation is lost in ring C due to the presence of dihydrogens at position 3. This makes the ring B out of plane. Also it reduces the absorption wavelength and is falls in the UV-A and UV-B region so that the molecule can be effectively used as UV filter in sun screen lotions and other photo protective cosmetics. The Table 1 describes the donor–acceptor interactions of the molecule. This is obtained from the NBO analysis by the Gaussian 09 software package [3], [4], [5]. The presence of hydrogen bonding between the carbonyl oxygen and H30 has been confirmed through this analysis. It shows that both the lone pair of electrons in carbonyl oxygen donates to the σ* orbital of O3–H30. The higher interaction energy indicates that the hydrogen bond is strong enough to restrict the breaking of O3–H30 bond to form a radical at position 5. This makes the bond dissociation energy (BDE) at this position higher than that at position 7 [6].

**Table 1:** Some important donor-accptor interactions in Eriodictyol.

	Donor NBO		Acceptor NBO	*E*(2) (Kcal/mol)	*E*(*j*)−*E*(*i*) (HF)	*F*(*I*,*j*) (HF)
σ	O6–H33	σ*	C20–C21	4.64	1.31	0.07
σ	O6–C21	σ*	C14–C19	1.6	1.47	0.044
σ	O6–C21	σ*	C15–C20	1.28	1.46	0.039
σ	O6–C21	σ*	C19–C21	0.67	1.43	0.028
σ	O6–C21	σ*	C20–C21	0.93	1.47	0.033
n1	O6	σ*	C19–C21	6.12	1.11	0.074
n2	O6	π*	C20–C21	28.03	0.33	0.091
σ	O5–H32	σ*	C19–C21	3.37	1.3	0.06
σ	O5–C19	σ*	C9–C14	1.31	1.45	0.039
σ	O5–C19	σ*	C14–C19	0.92	1.47	0.033
σ	O5–C19	σ*	C20–C21	1.73	1.47	0.045
n2	O5	σ*	O6–H33	2.65	1	0.046
n1	O5	σ*	C14–C19	5.87	1.17	0.074
n1	O5	σ*	C19–C21	0.58	1.13	0.023
n1	O5	π*	C14–C19	23.85	0.34	0.087
σ	O4–H31	σ*	C16–C18	3.92	1.3	0.064
σ	O4–C18	σ*	C12–C16	1.45	1.5	0.042
σ	O4–C18	σ*	C13–C17	1.35	1.48	0.04
σ	O4–C18	σ*	C16–C18	0.55	1.47	0.025
σ	O4–C18	σ*	C17–C18	0.69	1.48	0.029
n2	O4	σ*	C17–C18	5.75	1.14	0.072
n1	O4	π*	C16–C18	29.09	0.33	0.095
σ	O3–H30	σ*	C13–C17	4.72	1.3	0.07
σ	O3–C13	σ*	C10–C12	1.66	1.44	0.044
σ	O3–C13	σ*	C10–C13	0.82	1.43	0.031
σ	O3–C13	σ*	C13–C17	0.97	1.47	0.034
σ	O3–C13	σ*	C17–C18	1.46	1.47	0.042
n2	O3	σ*	C10–C13	7.16	1.06	0.078
n1	O3	σ*	C13–C17	0.51	1.1	0.021
n1	O3	π*	C13–C17	38.64	0.31	0.103
σ	O2–C11	σ*	O3–H30	0.57	1.42	0.026
σ	O2–C11	σ*	C7–C8	0.58	1.37	0.025
σ	O2–C11	σ*	C8–C11	0.86	1.41	0.031
σ	O2–C11	σ*	C10–C11	1.25	1.52	0.039
σ	O2–C11	σ*	C10–C12	1.51	1.55	0.044
π	O2–C11	σ*	C7–C8	0.71	0.71	0.02
π	O2–C11	σ*	C8–H23	1.68	0.79	0.033
π	O2–C11	σ*	C10–C12	4.84	0.37	0.043
π*	O2–C11	σ*	C8–H23	1.2	0.44	0.056
π*	O2–C11	π*	C10–C12	117.88	0.02	0.076
n1	O2	σ*	O3–H30	4.33	1.05	0.061
n1	O2	σ*	C10–C11	5.34	1.15	0.07
n2	O2	σ*	O3–H30	23.76	0.71	0.118
n2	O2	σ*	C7–C8	0.54	0.66	0.017
n2	O2	σ*	C8–C11	16.74	0.7	0.099
n2	O2	σ*	C10–C11	8.94	0.81	0.077

## 2 Materials and method

The stable conformer (lowest energetic) obtained from the potential energy scanning (PES) of Eriodictyol has been optimized with DFT-B3LYP/6–31G+(d, p) [7], [8], [9]. The structure with numbering is shown in Fig. 1. The energy gap of the stable conformer and the related global reactive descriptors has been computed. The TDDFT tool [10] in Gaussian 09 software package has been implemented to study the excited state characteristics of the molecule and has found that the molecule can be used as an effective UV filter since it absorption falls in the UV-A–UV-B region. The results are confirmed by the combined NBO–NLMO analysis [10], which is done though Gaussian 09 software package. Besides this the mechanistic evaluation shows that the hydrogen atom transfer mechanism is suitable to explain the radical scavenging activity of eriodictyol and the position 3′ is the most reactive site in it. These are further confirmed by the charge analysis *via* Natural Population Analysis (NPA), pKa value [11] and bond order analysis. The donor acceptor interaction energy results from NBO have been given Table 1 and it confirms the presence of hydrogen bond between the carbonyl oxygen and the H30 at position 5.
